# Assessing the SAfety and FEasibility of bedside portable low-field brain Magnetic Resonance Imaging in patients on ECMO (SAFE-MRI ECMO study): study protocol and first case series experience

**DOI:** 10.1186/s13054-022-03990-6

**Published:** 2022-04-30

**Authors:** Sung-Min Cho, Christopher Wilcox, Steven Keller, Matthew Acton, Hannah Rando, Eric Etchill, Katherine Giuliano, Errol L. Bush, Haris I. Sair, John Pitts, Bo Soo Kim, Glenn Whitman

**Affiliations:** 1 Division of Cardiac Surgery, Department of Surgery, Johns Hopkins University School of Medicine, 600 N. Wolfe Street, Phipps 455, Baltimore, MD 21287 USA; 2 Neuroscience Critical Care Division, Departments of Neurology, Neurosurgery, and Anesthesiology and Critical Care Medicine, Johns Hopkins University School of Medicine, Baltimore, MD USA; 3 Department of Biomedical Engineering, Johns Hopkins University School of Medicine, Baltimore, USA; 4 Division of Pulmonary and Critical Care Medicine, Department of Medicine, Johns Hopkins University School of Medicine, Baltimore, USA; 5 Division of Thoracic Surgery, Department of Surgery, Johns Hopkins University School of Medicine, Baltimore, USA; 6 Division of Neuroradiology, Department of Radiology and Radiological Science, Johns Hopkins University School of Medicine, Baltimore, USA; 7 Hyperfine, Guilford, CT USA

**Keywords:** Portable MRI, ECMO, Safety, Bedside, Neuroimaging, Brain

## Abstract

**Background:**

To assess the safety and feasibility of imaging of the brain with a point-of-care (POC) magnetic resonance imaging (MRI) system in patients on extracorporeal membrane oxygenation (ECMO). Early detection of acute brain injury (ABI) is critical in improving survival for patients with ECMO support.

**Methods:**

Patients from a single tertiary academic ECMO center who underwent head CT (HCT), followed by POC brain MRI examinations within 24 h following HCT while on ECMO. Primary outcomes were safety and feasibility, defined as completion of MRI examination without serious adverse events (SAEs). Secondary outcome was the quality of MR images in assessing ABIs.

**Results:**

We report 3 consecutive adult patients (median age 47 years; 67% male) with veno-arterial (*n* = 1) and veno-venous ECMO (*n* = 2) (VA- and VV-ECMO) support. All patients were imaged successfully without SAEs. Times to complete POC brain MRI examinations were 34, 40, and 43 min. Two patients had ECMO suction events, resolved with fluid and repositioning. Two patients were found to have an unsuspected acute stroke, well visualized with MRI.

**Conclusions:**

Adult patients with VA- or VV-ECMO support can be safely imaged with low-field POC brain MRI in the intensive care unit, allowing for the assessment of presence and timing of ABI.

**Supplementary Information:**

The online version contains supplementary material available at 10.1186/s13054-022-03990-6.

## Introduction

Despite rapidly expanding use [1], the therapeutic potential of ECMO is limited by significant morbidity such as acute brain injury (ABI), a devastating complication [2, 3]. Protocolized neuromonitoring improves the detection of ABI and may limit its sequelae with timely management [4]. However, early diagnosis of ABI is difficult due to an inability to safely transport patients and lack of available transport personnel. Even when possible, HCT (currently the only imaging modality used) has limited sensitivity for detecting ischemic ABI. The gold standard for diagnosing ABI, most commonly used conventional systems rely on high-strength magnetic fields (1.5-3T), is incompatible with extracorporeal life support circuits and equipment due to safety concerns including heating, migration, and malfunction.

Recent advances in low-field, portable MRI technology enable acquisition of clinically meaningful imaging in the presence of ferromagnetic materials [5]. Low-field MRI (0.064T) produces less than 5 Gauss beyond the 5-foot safety zone of the magnet’s center. This is a marked reduction in the magnetic footprint compared with conventional MRI, making it feasible for ICU use without adverse events [5]. Phantom and animal studies demonstrate the safety and compatibility of portable MRI without deleterious magnetic force or heating of the ECMO circuit or its components. [6]

The current study seeks (1) to evaluate the safety and feasibility of low-field point-of-care (POC) MRI to obtain brain imaging in ECMO patients and (2) to use those MRI images to assess for the presence of ABI. We hypothesized that low-field MRI can produce high-quality neuroimages superior to HCT to enable bedside detection of ABI.

## Methods

The Johns Hopkins Medicine Institutional Review Board approved this study (IRB00285716). Consent was obtained from a legally authorized representative as enrolled patients were unable to provide consent.

### Study design/population

This study presents the first 3 patients of an ongoing prospective observational study of POC MRI in adults with VA- or VV-ECMO. Exclusion criteria included weight over 200 kg, active pregnancy, and contraindications to 1.5T MRI other than ECMO such as incompatible implants. ABI included anoxic brain injury, ischemic stroke, and hemorrhagic stroke.

### Study procedure

HCT examinations were routinely performed on these patients as part of a standardized neuromonitoring protocol [4], with the addition of POC MRI within 24 h utilizing a 64mT Swoop® MR imaging system (Hyperfine, Inc., Guilford, CT). The MR system was positioned into the patient’s room with all ICU equipment outside the magnet’s 5G safety line (Additional file 1: Figs. S1 and S2). Once the patient’s bed was aligned with the MRI head coil, 4 trained individuals, including a perfusionist, respiratory therapist, intensivist, and nurse, slid the patient into position using a lift-and-slide maneuver. Pads placed around the patient’s head prevented any motion. Vital signs were monitored continuously during the examination. Resultant images were read by a neuroradiologist (H.I.S.) who was blinded in clinical information.

Medical equipment was positioned appropriately to allow the portable MRI to be situated at the head of the bed. The patient was maintained as flat as possible while positioning, at the direction of the team leader. A research coordinator and intensivist physician monitored vital signs, ECMO flow, and positioning of the cannula and the endotracheal tube.

### Serious adverse events (SAEs)

The following changes were considered SAEs: (i) change in mean arterial pressure (MAP) of ± 20%; (ii) decrease in ECMO flow rate of 10%; or (iii) decrease in oxygen level (SpO_2_) of 10% from baseline.

### Outcomes

Primary outcomes were safety and feasibility, defined as completion of the POC MRI examination without SAEs. The secondary outcome was the quality of MR images compared to HCT images, determined by a board-certified neuroradiologist (H.I.S.).

## Results

### Patient characteristics

We report the first 3 consecutive patients (median age 47 years; 67% male) who had both HCT and POC MRI examinations performed during ECMO support. Two patients were supported with VV-ECMO due to COVID-19 acute respiratory syndrome (ARDS), and one was supported with VA-ECMO due to cardiogenic shock from massive pulmonary embolism (Table 1).

**Table 1:** Patient characteristics and adverse events during portable brain MRI scan

	Age (years)	Sex	BMI	ECMO indication	Cannulation strategy	Neurologic symptoms	MRI time (min)	HCT finding	MRI finding	ABI management	Adverse events*
1	47	Male	30.5	COVID-19 ARDS	Fem-IJ (VV-ECMO)	Coma under sedation	40	Acute left occipital ischemic stroke with hemorrhagic conversion	Acute left occipital ischemic stroke	Anticoagulation held and restarted with serial imaging studies	One self-limited ECMO suction event
2	45	Male	32.5	COVID-19 ARDS	Fem-IJ (VV-ECMO)	Coma under sedation	43	No acute findings	None	Not applicable	None
3	55	Female	27.1	Cardiogenic shock with PE	Fem–Fem (VA-ECMO)	Coma under sedation	34	No acute findings	Acute right basal ganglia ischemic infarct	Anticoagulation held and restarted with serial imaging studies	Frequent ECMO suction events, received intravenous fluids; improved with repositioning patient*

ARDS: Acute respiratory distress syndrome; VV: venovenous; Fem-IJ: femoral–internal jugular; VA: venoarterial; Fem-Fem: femoral–femoral; ECMO: extracorporeal membrane oxygenation; PE: pulmonary embolism; ABI: acute brain injury. *It was noted that the patients torso was positioned in slight extension, bed and MRI were flattened to remove extension with resolution in suction events. *There were no serious adverse events in all patients

### Safety and feasibility

All patients were imaged successfully, without significant changes in MAP, ECMO flow, and SpO_2_ (SAEs). MRI examinations were successfully completed in all three patients in 34, 40 and 43 min. Two patients had ECMO cannula suction events, which resolved with fluid bolus and repositioning (Table 1).

### Clinical presentation and imaging findings

**Fig. 1:**
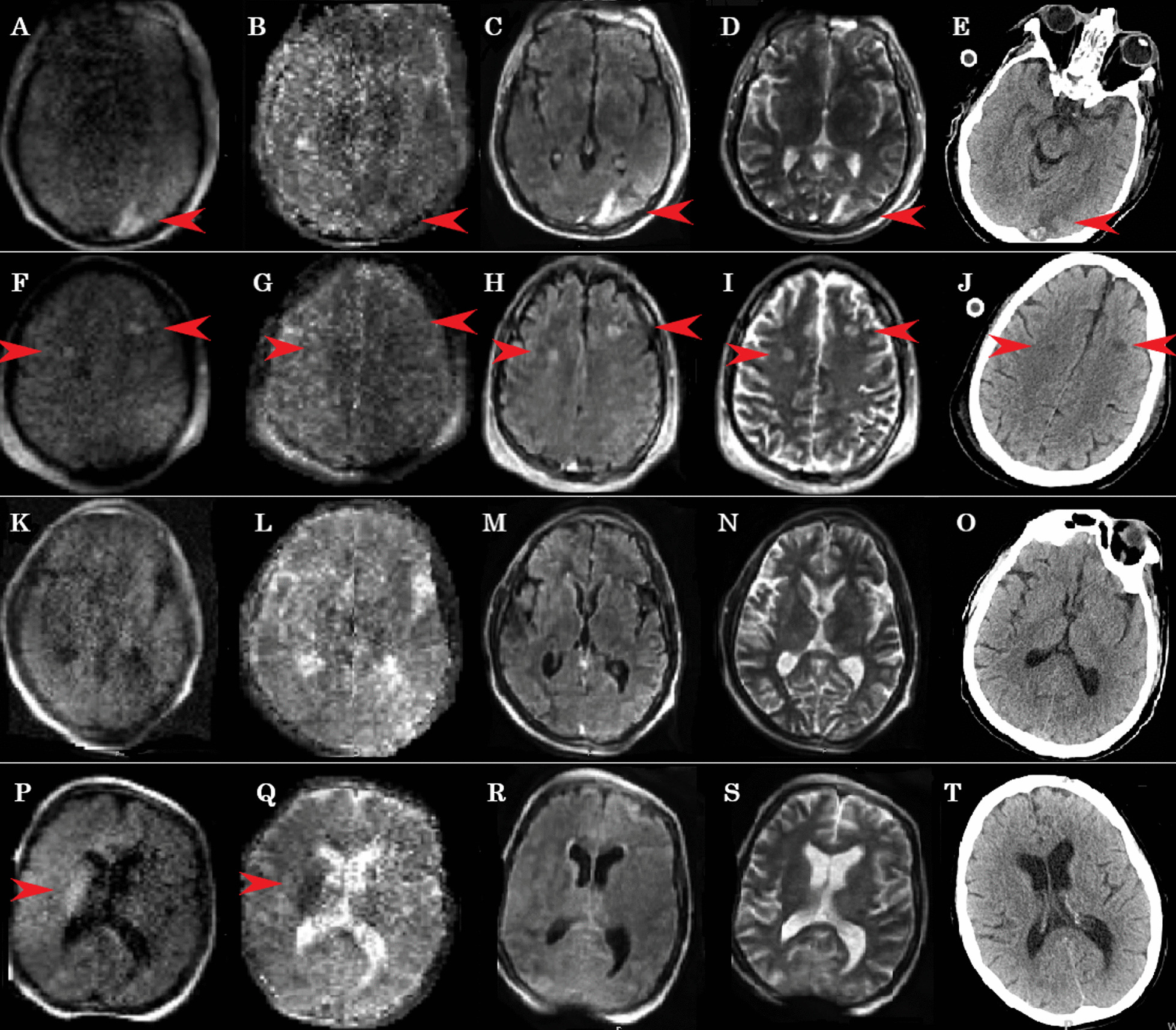
Point-of-care brain MRI images and corresponding CT images. Images **A**, **F**, **K**, **P** are diffusion-weighted imaging MRI, images **B**, **G**, **L**, **Q** are apparent diffusion coefficient MRI, images **C**, **H**, **M**, **R** are fluid-attenuated inversion recovery MRI, and images **E**, **J**, **O**, **T** are CT scans. Patient 1 (Images **A**–**J**) demonstrated acute occipital stroke with hemorrhagic conversion as well as two acute frontal lobe strokes are denoted by red arrows. Patient 2 (**K**–**O**) had normal MRI and CT scan. Patient 3 (**P**–**T**) had an acute right basal ganglia stroke (red arrow) confirmed on other MRI sequences and not identified on CT. Entire series of MRI sequences and images for these patients are available as the link to Digital Imaging and Communications in Medicine (DICOM) in the Hyperfine Cloud (Additional file 1: Appendix)

Patient #1: A 47-year-old male without significant medical history, cannulated with VV-ECMO for refractory COVID-19 ARDS. Patient had a routine HCT at day 15 of ECMO showing an acute ischemic stroke in the left occipital lobe with small hemorrhagic conversion. Ischemic stroke was confirmed with portable MRI; however, it was more difficult to diagnose small hemorrhagic conversion with MRI vs. HCT (Fig. 1, Additional file 1: Appendix).

Patient #2: A 45-year-old male without a significant medical history, cannulated with VV-ECMO for severe COVID-19 ARDS. Routine HCT at day 12 was conducted as our standard neuromonitoring protocol, followed by a POC MRI, neither of which showed any acute intracranial abnormalities.

Patient #3: A 55-year-old female with a recent pulmonary right upper lobectomy for primary lung adenocarcinoma with massive pulmonary embolism postoperatively, who required VA-ECMO for right heart failure. Although HCT was normal at day 4, the subsequent POC MRI within 24 h showed an acute right basal ganglia ischemic stroke.

### Quality of images

MR images were able to detect discernible pathologies with good quality. Two patients had acute strokes (#1, #3), which were well visualized by POC MRI, one of which was not seen on HCT (patient #3).

## Discussion

This study evaluated the feasibility and safety of portable POC brain MRI in patients with ECMO support, which represents a “first-in-human” investigation of this technology in the ECMO setting. POC MRI was shown to be safe and feasible with all examinations completed in under 43 min without SAEs. In ECMO patients where MRI at 1.5T is contraindicated and transportation for HCT can be dangerous and requires significant human resources, POC MRI allows early ABI detection, which is critical in improving ECMO survival [4]. As demonstrated, POC MRI detected new ischemic strokes, one of which was not visualized with HCT, suggesting that POC MRI may be a good bedside diagnostic tool for cerebral ischemia. As demonstrated in patient #1, the detection of hemorrhagic conversion in ischemic stroke needs further experience and validation, especially when hemorrhages are small.

This early experience provides insight into how to further improve care of ECMO patients by optimizing duration of the MRI examination time and patient positioning. In this study, four MR sequences were obtained in a relatively long scan time: T1-weighted, T2-weighted, fluid-attenuated inversion recovery, and diffusion-weighted imaging with an automatically calculated apparent diffusion coefficient map. Eliminating one or two of the sequences that are mirrored by others could decrease duration of the MRI examination, such as the T1. Patient body habitus, as seen in our patients, made positioning in the scanner challenging. As observed, 2 patients had temporary ECMO suction events that appeared to be the result of insufficient support of the thoraco-lumbar spine leading to flexion at the groin. Repositioning the body as flat as possible, thereby preventing flexion of the spine between the bed and the scanning platform, resolved the suction events, making it likely that the femoral drainage cannula position was compromised.

Overall, our study demonstrates that POC MRI in the ECMO patient is feasible and may offer logistic as well as diagnostic advantages over HCT, particularly given that early detection and timely intervention of ABI are of paramount importance in improving outcomes in the care of ECMO patients. Accessible POC MRI has the potential to markedly improve our ability to diagnose subclinical ABI and to immediately alter clinical management with the goal of mitigating injury. However, further research and experience with more patients is required to assess the “safety and feasibility” of POC MRI in ECMO because our study reported only 3 patients as a preliminary result.

## Conclusions

Adult patients with VA- and VV-ECMO support can be safely imaged with a low-field POC brain MR at the bedside in the ICU setting, allowing for the assessment and treatment of ABI.

## Supplementary Information

**Additional file 1**. Appendix.

## Data Availability

The authors agree to make the data available.
