# Supplementary material for: Assessing the SAfety and FEasibility of bedside portable low-field brain Magnetic Resonance Imaging in patients on ECMO (SAFE-MRI ECMO study): study protocol and first case series experience
Source: Crit Care. 2022 Apr 30;26:119. doi: 10.1186/s13054-022-03990-6 (PMC9059694; doi:10.1186/s13054-022-03990-6)
Supplement: Supplementary file 1 — Additional file 1. Appendix. [file 13054_2022_3990_MOESM1_ESM.docx]

**Supplemental Material**

**Supplemental Figure 1:** Patient with ECMO support within the Swoop® scanner.

ECMO was kept outside the scanner 5G line.


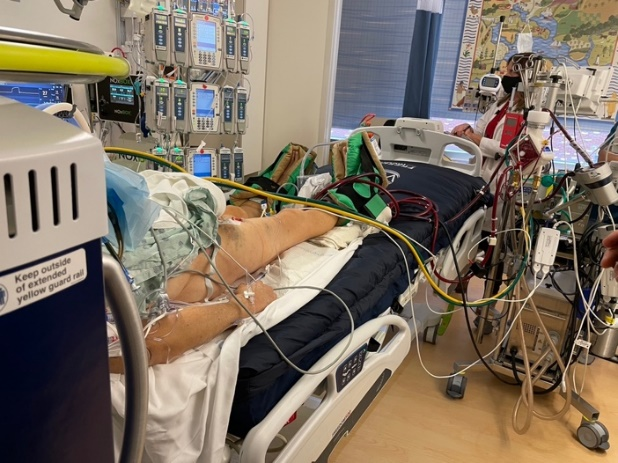


**Supplemental Figure 2:** (A) A model patient fully in position within the Hyperfine MRI device with the 5 gauss line shown by the extended yellow ring over the patient. Standard medical devices, such as the ventilator and IV pump on the right side of this model patient are safe and fully functional outside 5 gauss line. (B) Schematic of 5 gauss line and the strength of magnetic field.

**
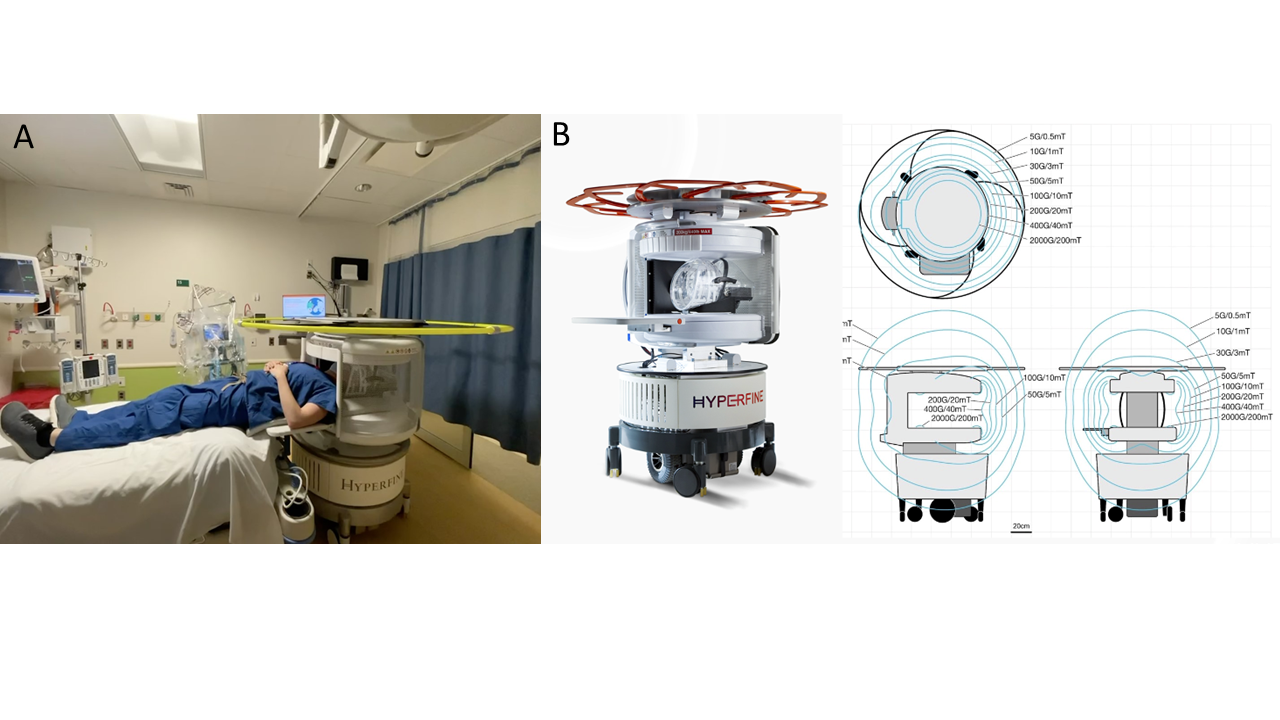
**

**Appendix:** MRI sequences and images for 3 ECMO patients as the link to Digital Imaging and Communications in Medicine (DICOM) in the Hyperfine Cloud.

| Patient | Link to Hyperfine Cloud Images (DICOM) |
| --- | --- |
| #1 | <https://viva.purview.net/instantshare.php?jwt=enc01-n26ZJ0y84MkELxuA0XEC1i9y_l3BBdXx-A52yGp8DfiyA6ofM5SknBvL8fWGooQS> |
| #2 | <https://viva.purview.net/instantshare.php?jwt=enc01-DE0UCIQB0_5bWVROziMpelKzSmrXjl9oeZIwyZ5KdGZlZnof17yVDLPi0OEyyH-s> |
| #3 | <https://viva.purview.net/instantshare.php?jwt=enc01-R0WN9D0HQ6q-FxiM-7knZIJMHHN3snj5KYcXU1-mptUU-qWOiK0s0l5jEv3zf5x4> |
